# Persulfate Activation by Cobalt-Doped Pyrite Nanoparticles for Oxidative Removal of 4-Chlorophenol

**DOI:** 10.3390/toxics14080663

**Published:** 2026-07-27

**Authors:** Mengyang Ni, Fangru He, Chuanjia Jiang, Hongyang Wang

**Affiliations:** 1 State Key Laboratory of Environmental Criteria and Risk Assessment, Chinese Research Academy of Environmental Sciences, Beijing 100012, China; 2 College of Environmental Science and Engineering, Nankai University, Tianjin 300350, China

**Keywords:** iron sulfide, peroxydisulfate, peroxymonosulfate, high-valent iron species, 4-chlorophenol

## Abstract

Persulfate-based Fenton-like oxidation is one of the most promising technologies for in situ chemical oxidation (ISCO) remediation of groundwater contamination, yet the mechanisms affecting persulfate activation efficiency remain underexplored. Herein, we investigated the efficiency and mechanisms of peroxydisulfate (PDS) and peroxymonosulfate (PMS) activation by cobalt-doped pyrite (Co-FeS_2_) nanoparticles for degradation of 4-chlorophenol (4-CP), a model groundwater contaminant. Notably, the degradation kinetics in the Co-FeS_2_/PDS system exhibited a unique “three-stage” characteristic, wherein the 4-CP degradation rate underwent a jump during the 3–5 min phase. This kinetic anomaly stems from the specific generation dynamics of ferryl species (Fe^IV^=O), which experienced a 3 min lag phase followed by a rapid burst. Theoretical calculations revealed that surface-accumulated SO_4_^2−^ reduces the thermodynamic energy barrier for Fe^IV^=O formation, which accounts for this rapid generation subsequent to the initial lag phase. Furthermore, while hydroxyl (•OH) and sulfate (SO_4_^•−^) radicals were critical in both systems, •OH concentration was higher than SO_4_^•−^ concentration in the Co-FeS_2_/PDS system, whereas the Co-FeS_2_/PMS system exhibited the reverse trend. Moreover, homogeneous persulfate activation mediated by dissolved Fe(II) contributed to 4-CP degradation, but to different degrees in the two systems. This study provides mechanistic insights into persulfate-based ISCO processes for groundwater remediation.

## 1. Introduction

The widespread contamination of subsurface environments by recalcitrant organic pollutants poses a severe threat to ecosystems and human health [1,2,3]. Over the last few decades, enormous efforts have been devoted to research on in situ remediation of soil and groundwater with organic pollutants via reduction [4,5,6], oxidation [7,8,9], hydrolysis [10,11,12], and biodegradation [13,14]. Among these approaches, in situ chemical oxidation (ISCO), which involves the injection and activation of chemical oxidants underground, has emerged as a highly promising remediation strategy for efficient degradation of a variety of organic contaminants, attributed to the generation of abundant reactive species [15,16,17]. Specifically, persulfates, including peroxymonosulfate (PMS) and peroxydisulfate (PDS), are predominantly favored for field-scale applications due to their remarkable longevity and extended transport capabilities in porous media [18,19,20].

The activation mechanisms of PMS and PDS typically involve the adsorption and dissociation of the persulfate ions on the catalyst surface, as well as charge transfer to the transition metal center, which leads to the cleavage of O-O bonds and the formation of active species such as hydroxyl radicals (•OH), sulfate radicals (SO_4_^•−^), singlet oxygen, and high-valent metal species [21,22,23]. However, due to structural differences among various oxidants and variations in O-O bond energies (ranging from 144 kJ/mol to 213 kJ/mol), there are significant differences in the adsorption configurations of different oxidants at the same active site and in the mechanisms of interfacial electron transfer. Consequently, activation pathways and efficiency exhibit considerable variation [24,25]. For example, under identical conditions, PMS exhibits higher reactivity than PDS in electrocatalytic systems [26]. In addition, the properties of the catalytic site itself also influence the radical formation pathways and efficiency during the activation of the oxidizing agent [27,28]. Previous studies have shown that electron-rich Fe sites in pyrite can promote the adsorption and cleavage of PMS, significantly lowering the energy barrier for electron transfer by lengthening the O-O bond; in contrast, electron-deficient magnetite lacks effective PMS adsorption sites, which hinders the generation of free radicals [29]. In-depth understanding of the interfacial interactions between different persulfates on the material surface will help elucidate the universal activation mechanisms of active sites in multi-oxidant systems.

This study systematically investigated the performance and mechanisms of cobalt-doped pyrite (Co-FeS_2_) for activating PMS and PDS to degrade 4-chlorophenol (4-CP), an aromatic chlorinated organic compound commonly found in groundwater [30,31]. Cobalt-doped FeS_2_ was selected as a model material due to its superior performance in activating oxidants, including PMS [32,33,34]. Quenching and probe experiments were conducted to reveal differences in reactive species generation for the two types of persulfates. The activation mechanisms of different systems were elucidated by analyzing the distribution of dissolved iron species and the evolution of the valence states of surface-bound iron. Finally, we explored the effects of coexisting matrix components (such as inorganic cations and anions) widely present in groundwater on the degradation efficiency of each reaction system. The findings can provide technical support for tailoring persulfate-based in situ remediation strategies to meet specific needs in complex real-world sites.

## 2. Materials and Methods

### 2.1. Preparation and Characterization of Material

Co-doped FeS_2_ (Co-FeS_2_) was synthesized via a solvothermal method [33,35], with details on the procedures and chemical reagents given in the Supplementary Materials. The material was characterized by X-ray diffraction (XRD, Ultima IV, Rigaku, Tokyo, Japan) and transmission electron microscopy (TEM, JEM-2800, JEOL, Tokyo, Japan) to determine its composition and morphology.

### 2.2. Persulfate Activation and 4-CP Degradation Experiment

Batch kinetic degradation experiments were conducted in a shaking incubator at 25 °C and 180 rpm. The Co-FeS_2_ nanoparticle suspension and the persulfate solution were prepared in separate flasks, using a 2 mM borate buffer to adjust the pH to 7.2. The reaction was initiated by rapidly mixing the persulfate solution with the Co-FeS_2_ suspension. At predetermined intervals, 0.2 mL aliquots of the reaction mixture were withdrawn and immediately quenched with an equal volume of sodium thiosulfate solution. The concentration of 4-CP was determined using high-performance liquid chromatography (Agilent 1260, Santa Clara, CA, USA). Detailed procedures are provided in the Supplementary Materials.

### 2.3. Detection and Quantification of Reactive Species

The steady-state concentrations of •OH and SO_4_^•−^ were calculated using competitive kinetic experiments, with benzoic acid (BA) and p-nitrobenzoic acid (*p*-NBA) used as probes. Methyl phenyl sulfoxide (PMSO) was used as a probe for ferryl species (Fe^IV^=O), and the formation of Fe^IV^=O was evaluated by calculating the efficiency of PMSO conversion to methyl phenyl sulfone (PMSO_2_) [36]. The detailed experimental procedures and the calculation methods for the steady-state concentrations of the radicals are provided in the Supplementary Materials, and the conditions for the HPLC analysis are listed in Table S1.

### 2.4. Determination of Fe(II) and Total Fe Concentrations in Solution

The concentration of dissolved Fe(II) was determined using the 1,10-phenanthroline colorimetric method. Total iron content was quantified by reducing Fe(III) to Fe(II) using hydroxylamine hydrochloride. The detailed experimental procedures are provided in the Supplementary Materials.

### 2.5. Density Functional Theory (DFT) Calculation

Theoretical calculations were employed to investigate the effects of SO_4_^2−^ on the Fe^IV^=O generation during PDS activation by Co-FeS_2_. Details about the DFT calculation are provided in the Supplementary Materials.

## 3. Results and Discussion

### 3.1. 4-CP Degradation Performance

The Co-doped FeS_2_ was successfully synthesized using a simple solvothermal method, with Co accounting for approximately 2.45 wt% of the total mass of the material [33]. The TEM images (Figure S1) and XRD pattern (Figure S2) of the as-synthesized material were consistent with those for the Co-doped FeS_2_ materials reported in our previous study [33], and the spherical nanoparticles are of the pyrite phase (PDF#97-000-0316), with an average diameter of 137 nm [33].

**Figure 1:**
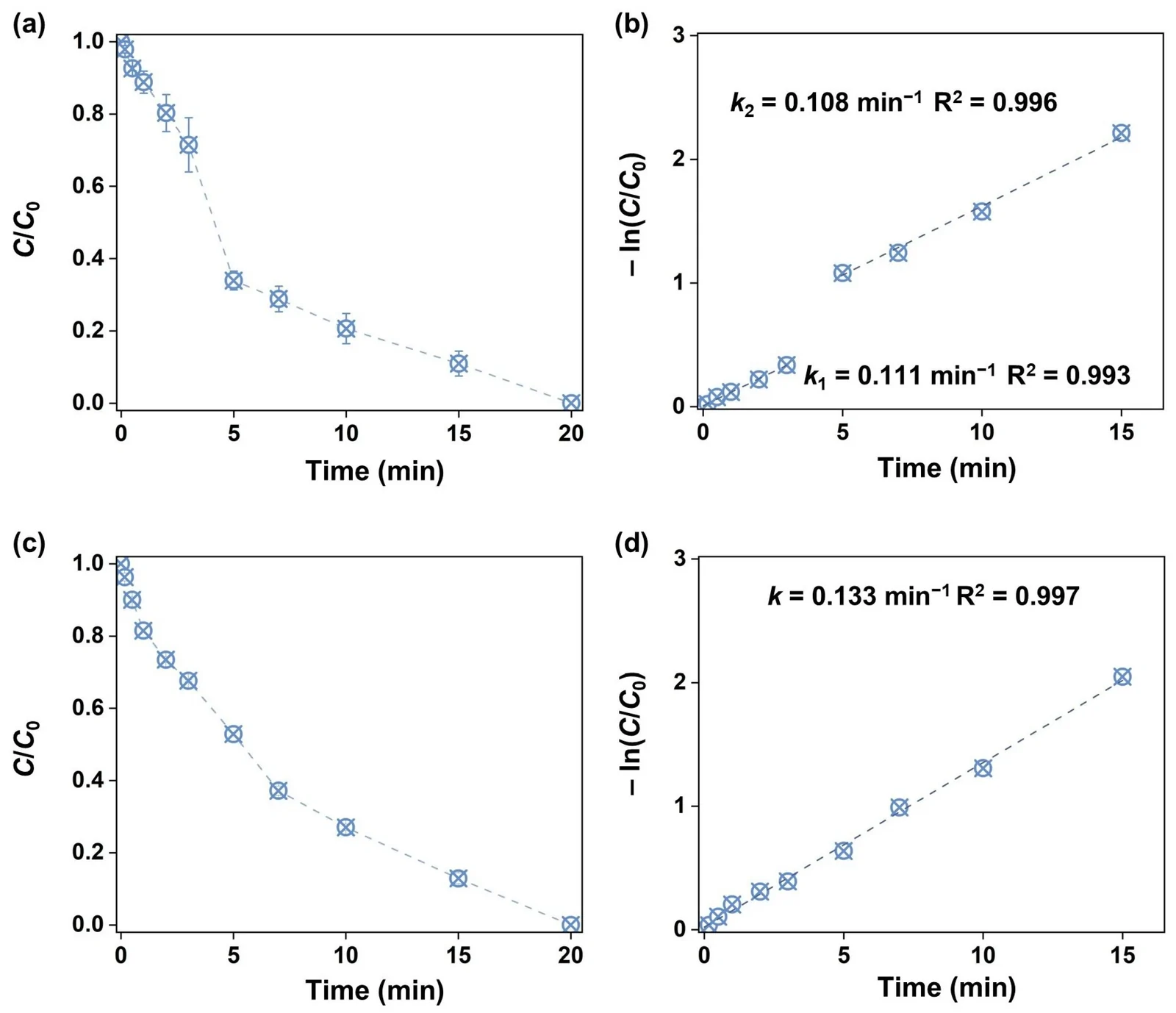
The 4-CP degradation curves (**a**,**c**) and kinetic fitting curves (**b**,**d**) via the activation of (**a**,**b**) PDS and (**c**,**d**) PMS by Co-FeS_2_. Initial 4-CP concentration: 0.1 mmol/L. (**a**,**b**) PDS dosage: 10 mmol/L, Co-FeS_2_ dosage: 0.2 g/L; (**c**,**d**) PMS concentration: 1 mmol/L, Co-FeS_2_ dosage: 0.1 g/L.

The Co-FeS_2_ material exhibited high efficiency for degradation of 4-CP via activation of both PDS and PMS, but the kinetic characteristics differed between the two types of persulfates. In the Co-FeS_2_-mediated PDS activation (Co-FeS_2_/PDS) system, nearly 100% removal of 4-CP was achieved within 20 min (Figure 1a). It is worth noting that the degradation of 4-CP followed a three-stage pattern. Specifically, during the initial reaction phase (0–3 min), the system achieved a 28.5% removal rate of 4-CP; however, within the extremely short time window of 3 to 5 min, the degradation of 4-CP accelerated sharply, and the removal rate surged to 62.7% during this stage; Subsequently, during the 5–20 min phase, the degradation rate leveled off, and the remaining 4-CP was removed relatively slowly. To further elucidate the reaction mechanism of this system, stepwise kinetic fitting was performed on the degradation process of 4-CP. The results indicate that the degradation of 4-CP followed a pseudo-first-order kinetic model in the first and third intervals (R^2^ > 0.99) (Figure 1b). Specifically, the rate constants of 4-CP degradation during the initial 0–3 min and the 5–15 min intervals were 0.111 min^−1^ and 0.108 min^−1^, respectively, suggesting a similar mechanism during the two stages (the slight decrease in the rate constant during the later stage may be attributed to the reduced concentration of the oxidizing agent in the system). Notably, the anomalous and significant jump in the reaction kinetics during the 3–5 min phase suggests that different types of active species were involved in the degradation of 4-CP at different stages of the reaction.

The Co-FeS_2_-mediated PMS activation (Co-FeS_2_/PMS) system also exhibited high 4-CP degradation efficiency, despite a low PMS concentration of 1 mmol/L (only one-tenth that of the oxidant in the Co-FeS_2_/PDS system) and a Co-FeS_2_ dosage of 0.1 g/L (only half the dosage of the Co-FeS_2_/PDS system), achieving nearly 100% removal of 4-CP within 20 min (Figure 1c). In contrast to the Co-FeS_2_/PDS system, the 4-CP degradation kinetics in the Co-FeS_2_/PMS system followed a pseudo-first-order model throughout the 20 min, with a rate constant of 0.133 min^−1^, and exhibited no staged changes (Figure 1d). This discrepancy suggests that the generation kinetics of reactive species differ between the two systems.

### 3.2. Analysis of Reactive Species During Persulfate Activation

**Figure 2:**
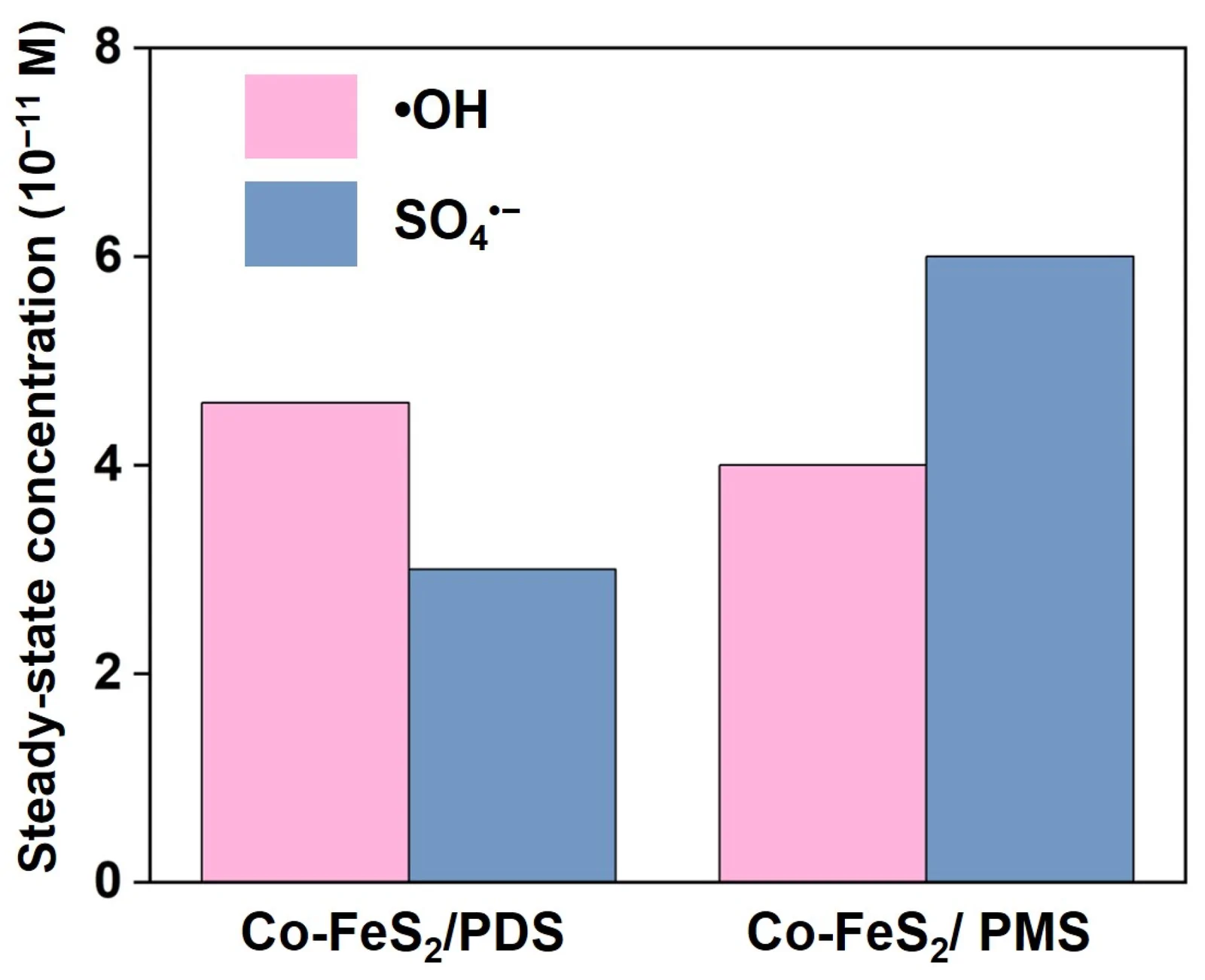
Steady-state concentrations of hydroxyl (•OH) and sulfate (SO_4_^•−^) radicals.

Benzoic acid and *p*-nitrobenzoic acid were used as probes to determine the steady-state concentrations of •OH and SO_4_^•−^, two reactive oxygen species (ROS) that are known to play important roles in pollutant degradation via persulfate activation [37,38]. Based on their degradation kinetics data (Figures S3 and S4), the steady-state concentrations of •OH and SO_4_^•−^ in each system were calculated (Figure 2). There is a significant difference in the dominant radical species between the Co-FeS_2_/PDS and Co-FeS_2_/PMS systems. Specifically, in the Co-FeS_2_/PDS system, the concentration of •OH is higher than that of SO_4_^•−^, whereas in the Co-FeS_2_/PMS system, SO_4_^•−^ is the dominant species.

**Figure 3:**
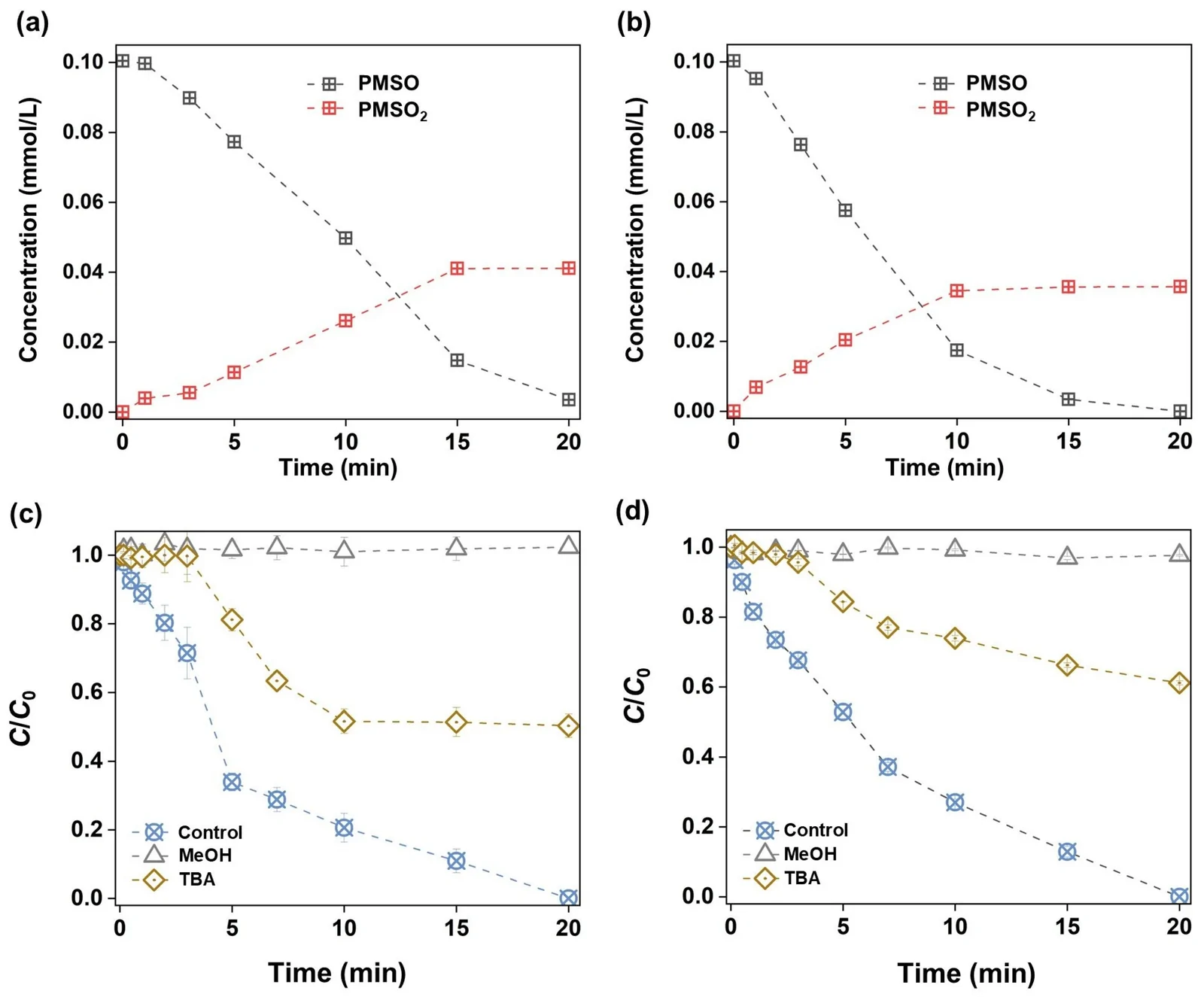
Curves of PMSO removal and PMSO_2_ formation in the (**a**) Co-FeS_2_/PDS and (**b**) Co-FeS_2_/PMS systems. Degradation curves of 4-CP with or without different quenchers (MeOH and TBA) in the (**c**) Co-FeS_2_/PDS and (**d**) Co-FeS_2_/PMS systems.

Apart from the ROS, high-valent metal species such as Fe^IV^=O may also play important roles in pollutant degradation via persulfate activation by metal-based materials [39,40,41]. The relevant abundance of Fe^IV^=O in the Co-FeS_2_/PMS and Co-FeS_2_/PDS systems was analyzed using PMSO as a probe. PMSO can be selectively oxidized by Fe^IV^=O via an oxygen transfer mechanism to form the corresponding sulfone (PMSO_2_), which differs significantly from its oxidation by ROS. Therefore, an analysis of the removal rate of PMSO and the formation rate of PMSO_2_ can be used to estimate the relevant abundance of Fe^IV^=O in the persulfate activation systems. As shown in Figure 3a,b, the formation of PMSO_2_ was detected in both the Co-FeS_2_/PMS and Co-FeS_2_/PDS systems, with conversion rates of PMSO to PMSO_2_ of 35.6% and 41.1%, respectively. Note that PMS can directly oxidize PMSO to PMSO_2_, leading to an overestimation of the Fe^IV^=O concentration, while no noticeable direct oxidation of PMSO by PDS was observed [42]. Therefore, the above results indicate that the Fe^IV^=O yield in the Co-FeS_2_/PDS system was higher than that in the Co-FeS_2_/PMS system. Notably, in the Co-FeS_2_/PDS system (Figure 3a), the concentration of PMSO_2_ increased slowly during the first 3 min and at a higher rate after 3 min, indicating that the formation of Fe^IV^=O in this system was latent during the initial reaction stage. By contrast, the concentration of PMSO_2_ in the Co-FeS_2_/PMS system showed a steady increase between 0 and 10 min (Figure 3b), indicating that the formation rate of Fe^IV^=O remains relatively stable throughout the process.

Quenching experiments were conducted to further identify the contribution of different active species to 4-CP degradation in the Co-FeS_2_/PMS and Co-FeS_2_/PDS systems. Methanol (MeOH) reacts rapidly with •OH and SO_4_^•−^ (*k*_[MeOH][•OH]_ = 1.0 × 10^9^ M^−1^ s^−1^, *k*_[MeOH][SO4•−]_ = 1.0 × 10^7^ M^−1^ s^−1^) [43], while also exhibiting a certain scavenging capacity for Fe^IV^=O (*k*_[MeOH][FeIV=O]_ = 2.5 × 10^3^ M^−1^ s^−1^) [44]. TBA can also effectively scavenge •OH (*k*_[TBA][•OH]_ = 5.9 × 10^8^ M^−1^ s^−1^) and, with a lower efficiency, SO_4_^•−^ (*k*_[TBA][SO4•−]_ = 8.0 × 10^5^ M^−1^ s^−1^), but it hardly reacts with Fe^IV^=O (*k*_[TBA][FeIV=O]_ = 6.0 × 10^1^ M^−1^ s^−1^) [43,45]. In the Co-FeS_2_/PDS system, the addition of MeOH completely suppressed the degradation of 4-CP (Figure 3c). However, the inhibitory effect of TBA exhibited distinct temporal patterns: during the early (0–3 min) and late (10–20 min) stages of the reaction, the degradation of 4-CP was essentially halted; however, during the 3–10 min stage, significant degradation of 4-CP still occurred. Therefore, it is speculated that in the initial stage, the degradation of 4-CP was primarily driven by •OH and SO_4_^•−^. However, after 3 min, Fe^IV^=O was rapidly generated in the system and participated in the oxidation process. In the Co-FeS_2_/PMS system, MeOH completely inhibited the degradation of 4-CP, whereas the addition of TBA significantly suppressed the degradation of 4-CP, though the 4-CP concentration continued to decrease throughout the process (Figure 3d). This observation indicates that some active species, which are likely a fraction of SO_4_^•−^ not quenched by TBA and Fe^IV^=O, continued to participate in the degradation of 4-CP.

**Figure 4:**
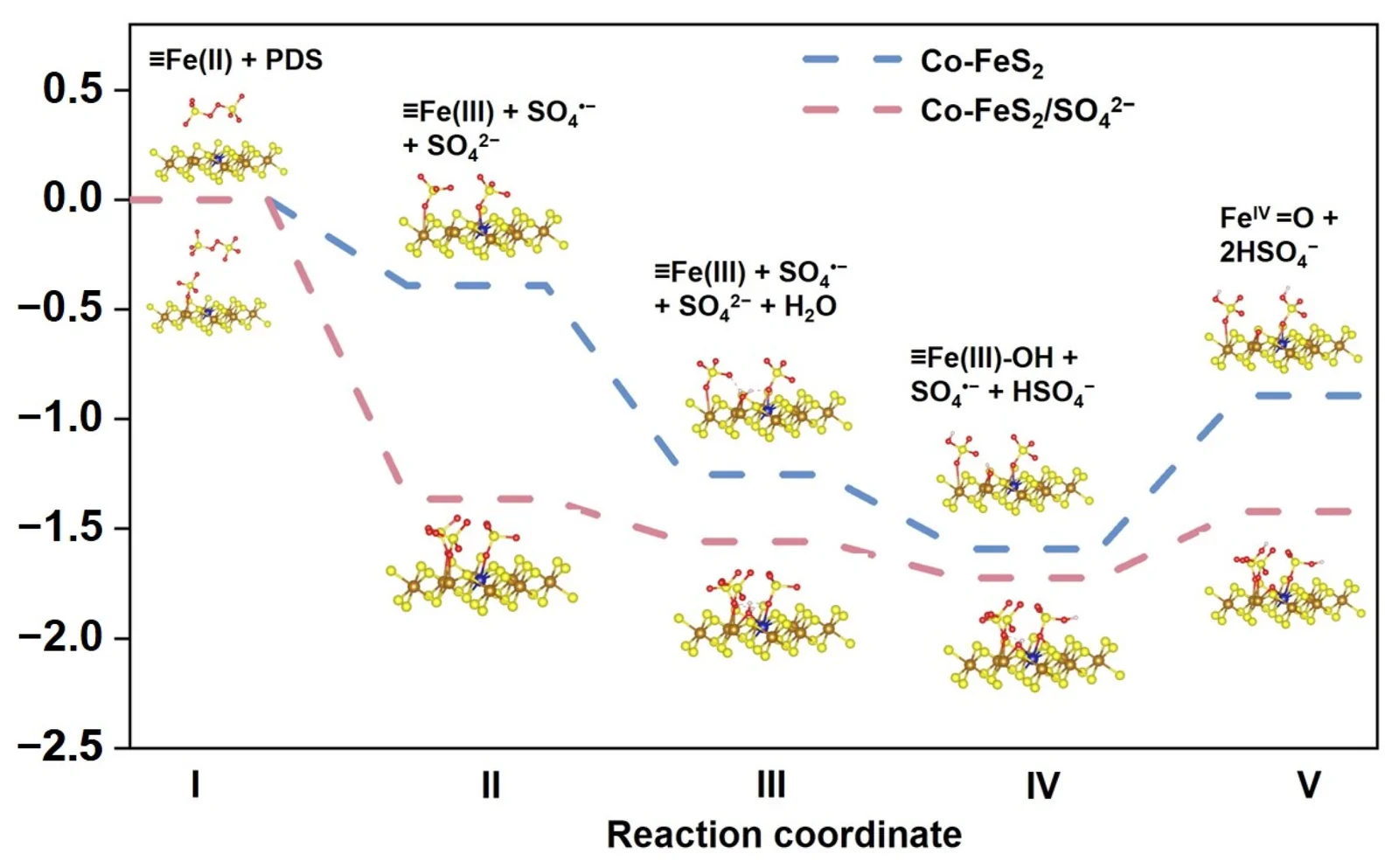
Changes in the free energy of the PDS activation pathway for Co-FeS_2_ in the presence (red line) and absence (blue line) of adsorbed SO_4_^2−^.

Furthermore, we investigated the mechanisms for the rapid generation of Fe^IV^=O in the PDS system during the 3–5 min reaction stage. Based on the literature, PDS first undergoes cleavage at the active site to generate SO_4_^•−^ and SO_4_^2−^, followed by electron transfer between SO_4_^•−^ and surface-adsorbed H_2_O to induce the formation of Fe^IV^=O [46]. Therefore, the enrichment of SO_4_^2−^ at the interface during the early reaction phase likely reduces the reaction energy barrier for the subsequent formation of Fe^IV^=O. To verify the above hypothesis at the atomic scale, we constructed DFT computational models based on the crystal structures of Co-FeS_2_ and PDS and calculated the free energy change along the PDS activation pathway for Co-FeS_2_ with or without adsorbed SO_4_^2−^. As shown in Figure 4, the peripheral O atoms (terminal oxygen) and central O atoms (bridge oxygen) of S_2_O_8_^2−^ are adsorbed by Fe and Co atoms, respectively, resulting in the formation of SO_4_^•−^ and SO_4_^2−^. This reaction proceeds spontaneously in the Co-FeS_2_ system, indicating that Co-FeS_2_ can effectively activate PDS to generate SO_4_^•−^. Subsequently, the remaining sites on the surface further adsorb and activate H_2_O, forming the Fe(III)-OH intermediate [46]. Finally, this intermediate interacts with SO_4_^•−^ to generate the Fe^IV^=O and HSO_4_^−^. Notably, in a microenvironment where SO_4_^2−^ is pre-adsorbed on the surface, the energy barrier (0.30 eV), which the Fe(III)-OH intermediate must overcome to react with SO_4_^•−^ and convert to Fe^IV^=O, is significantly lower than that in the Co-FeS_2_ system without adsorbed SO_4_^2−^ (0.70 eV). These theoretical calculation results indicate that the pre-accumulated SO_4_^2−^ on the material surface greatly reduces the reaction energy barrier, which may account for the rapid generation of Fe^IV^=O in the Co-FeS_2_/PDS system after 3 min.

### 3.3. Effects of Dissolved Fe(II) on Persulfate Activation and 4-CP Degradation

In persulfate activation by Fe-based materials, both Fe species on the surface of the materials and dissolved Fe(II) in the solution can mediate the generation of reactive species and pollutant degradation [47]. To elucidate the possible mechanisms by which dissolved Fe(II) may contribute to activation of the persulfates, we analyzed the dissolved Fe concentration as well as the speciation of surface Fe in the two systems.

**Figure 5:**
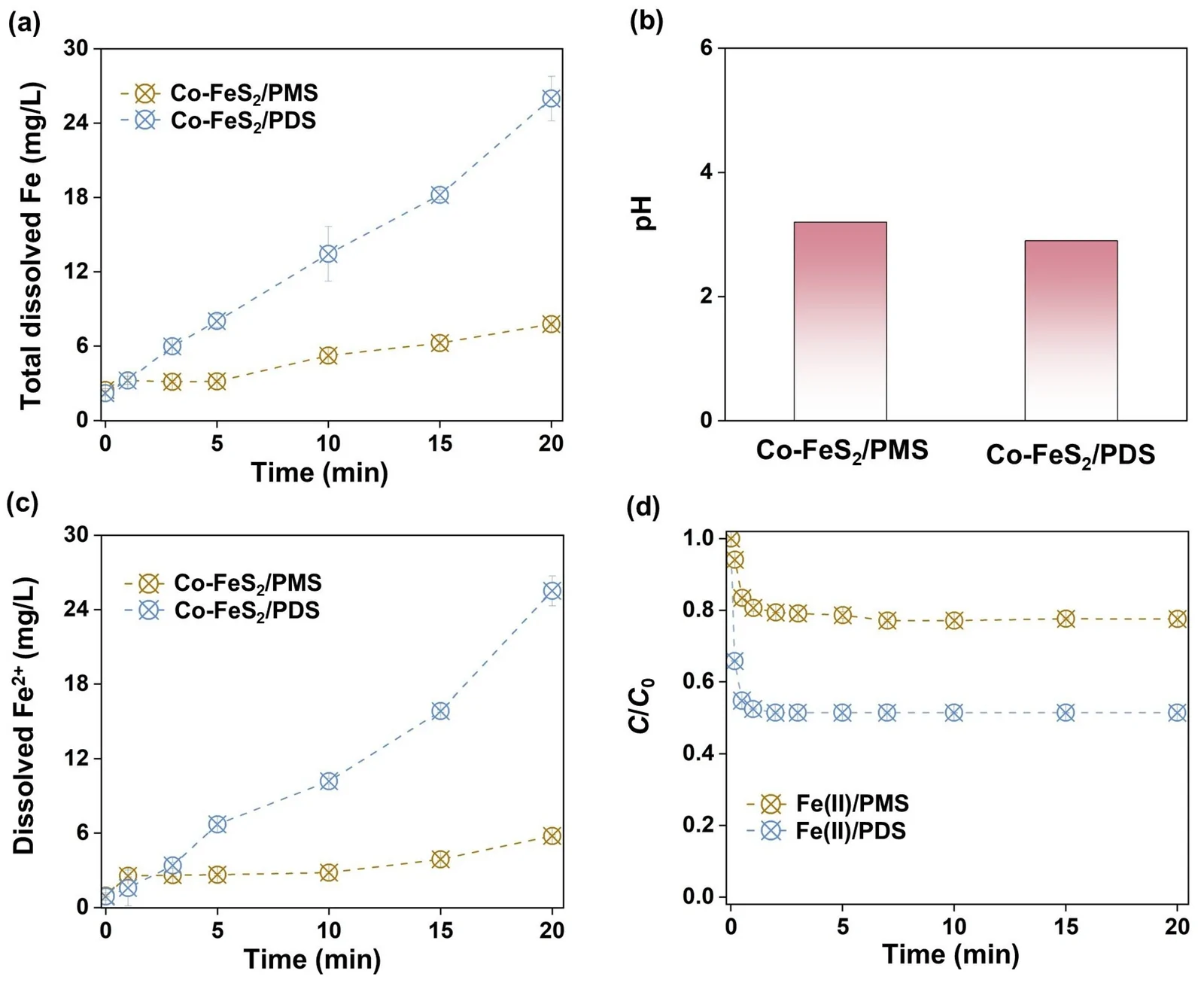
(**a**) Variations in total dissolved Fe concentration during the activation of PDS and PMS by Co-FeS_2_. (**b**) Solution pH after 20 min of reaction. (**c**) Variations in dissolved Fe(II) concentration during the activation of PDS and PMS by Co-FeS_2_, and (**d**) 4-CP degradation curves of the Fe(II)/PDS and Fe(II)/PMS systems.

We first measured dissolved Fe concentration in the systems during persulfate activation. In the Co-FeS_2_/PDS system, the concentration of total dissolved iron in the liquid phase continuously increased over the course of the reaction. After 20 min of reaction, the total iron leached into the solution reached as high as 27 mg/L (Figure 5a). The amount of dissolved Fe in the Co-FeS_2_/PMS system was significantly lower than that in the Co-FeS_2_/PDS system, with total dissolved Fe concentration reaching 7.9 mg/L after 20 min (Figure 5a). The higher Fe dissolution in the Co-FeS_2_/PDS system can be primarily attributed to the higher amount of Co-FeS_2_ in the Co-FeS_2_/PDS system (0.2 g/L) than that in the Co-FeS_2_/PMS system (0.1 g/L), as well as the pH difference between the two systems. Upon completion of the reaction, the pH values of the Co-FeS_2_/PMS and Co-FeS_2_/PDS systems were 3.2 and 2.9, respectively (Figure 5b). The difference in solution pH not only affected the oxidative dissolution of pyrite-based materials [48], but also influenced the distribution of dissolved iron species [49], especially their precipitation out of the aqueous solution phase [50].

An analysis of dissolved Fe(II) concentration in the reaction system revealed significant differences in the speciation of dissolved iron between the two systems (Figure 5c). After 20 min of reaction, the proportions of dissolved Fe(II) in the Co-FeS_2_/PDS and Co-FeS_2_/PMS systems reached 98.2% and 74.2%, respectively. These substantial fractions of dissolved Fe(II) were also expected to contribute to persulfate activation and 4-CP degradation. To test this hypothesis, we further conducted a 4-CP degradation test via homogeneous PDS or PMS activation mediated by dissolved Fe(II) ions (Figure 5d). The Fe(II) ion concentration was set to match the total Fe concentration leached at 20 min in the respective systems, and this amount of Fe(II) was introduced via a one-time addition to estimate the upper bound of the contribution from homogeneous persulfate activation. In these homogeneous systems, 4-CP was rapidly degraded within the first minute of the reaction, with degradation rates of 51% and 21%, respectively, for the Fe(II)/PDS and Fe(II)/PMS systems. Subsequently, the degradation curves plateaued as Fe(II) was rapidly and irreversibly oxidized to catalytically inactive Fe(III). The high 4-CP removal rate of 51% in the Fe(II)/PDS system indicates that, in the Co-FeS_2_/PDS system, both the Fe(II)-mediated homogeneous reaction and the heterogeneous reaction are important driving forces for 4-CP degradation. By contrast, the relatively low 4-CP removal rate of 21% in the Fe(II)/PMS system indicates that, in the Co-FeS_2_/PMS system, heterogeneous catalytic reactions on the material surface dominate the degradation of pollutants, while homogeneous reactions play a supporting role.

**Figure 6:**
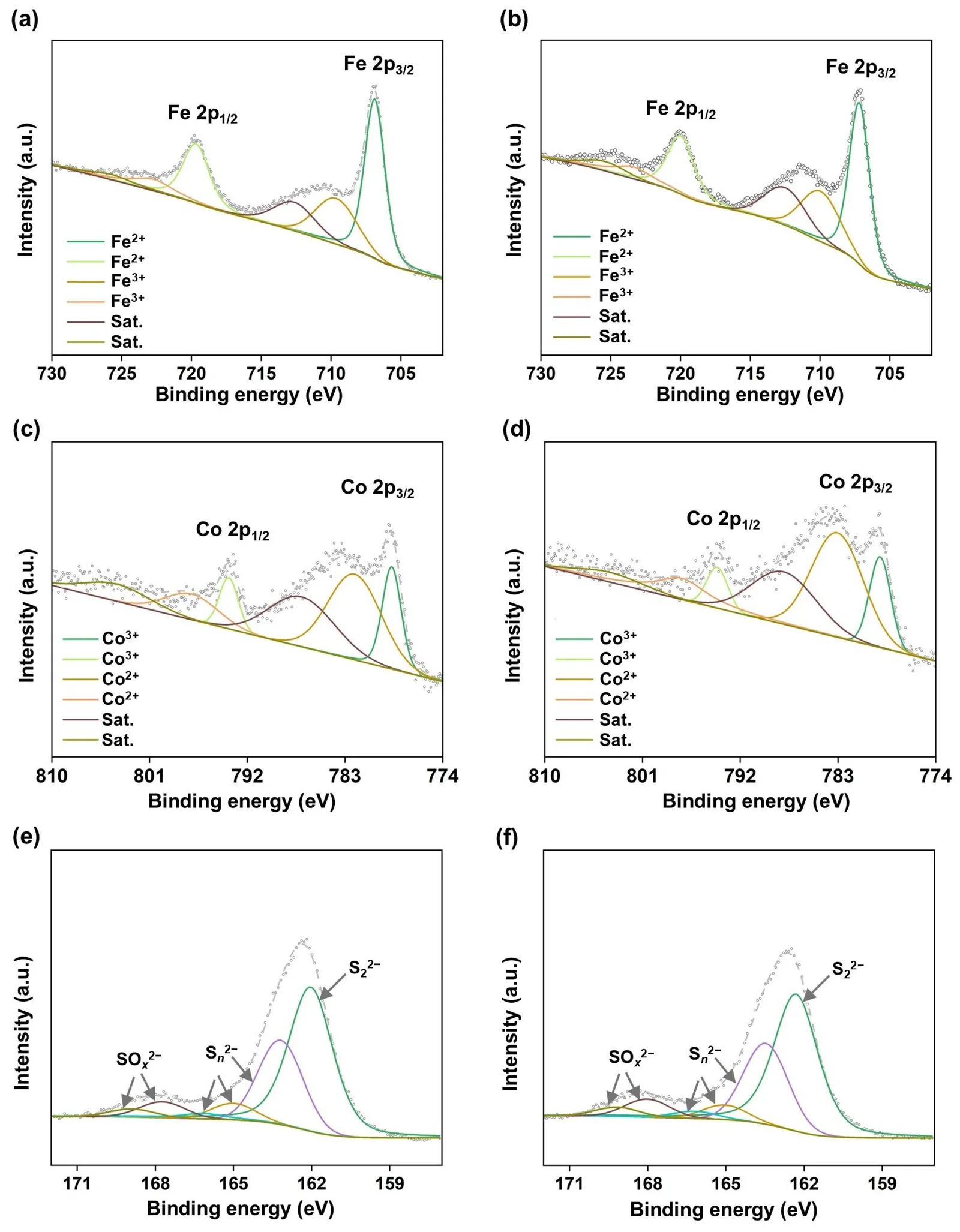
High-resolution XPS spectra of (**a**,**b**) Fe 2p, (**c**,**d**) Co 2p, and (**e**,**f**) S 2p after reaction in different systems: (**a**,**c**,**e**) Co-FeS_2_/PDS and (**b**,**d**,**f**) Co-FeS_2_/PMS.

The origin of the high abundance of Fe(II) in the solution phase was explored by XPS analysis of the materials after the reaction. In the high-resolution XPS spectrum of Fe 2p (Figure 6a,b), the fitted peaks at binding energies of 707 and 720 eV correspond to the Fe 2p_3/2_ and Fe 2p_1/2_ orbitals of Fe(II), respectively, while those at 710 and 723 eV correspond to the Fe 2p_3/2_ and Fe 2p_1/2_ orbitals of Fe(III), respectively [46]. In the high-resolution XPS spectrum of Co 2p (Figure 6c,d), the fitted peaks at binding energies of 779 and 794 eV correspond to the Co 2p_3/2_ and Co 2p_1/2_ orbitals of Co(III), respectively, while those at 783 and 797 eV correspond to the Co 2p_3/2_ and Co 2p_1/2_ orbitals of Co (II), respectively [51]. In the high-resolution XPS spectrum of S 2p, the fitted peak at 162 eV corresponds to the S_2_^2−^ species, those at 163, 165, and 166 eV correspond to S*_n_*^2−^, and those at 168 and 169 eV correspond to SO*_x_*^2−^ (Figure 6e,f) [52]. After the reaction, the proportion of Fe(II) on the surface of Co-FeS_2_ was 68.7% and 68.2%, respectively, for the Co-FeS_2_/PDS and Co-FeS_2_/PMS systems (Table S2). Similarly, Co(II) remained the dominant surface Co species after the reaction, accounting for 65.8% and 71.5% in the Co-FeS_2_/PDS and Co-FeS_2_/PMS systems, respectively (Table S3). However, the proportion of S_2_^2−^ on the surface of Co-FeS_2_ was only 55.8% and 55.6%, respectively, for the Co-FeS_2_/PDS and Co-FeS_2_/PMS systems (Table S4). Supported by previous studies, it can be inferred that the structural S(–I) of Co-FeS_2_ was consumed as an electron donor [53], thereby continuously reducing Fe(III) in both the solution and on the material surface to Fe(II), ultimately maintaining the high proportion of Fe(II) during the reaction.

### 3.4. Effects of Common Ions on 4-CP Degradation via Persulfate Activation

**Figure 7:**
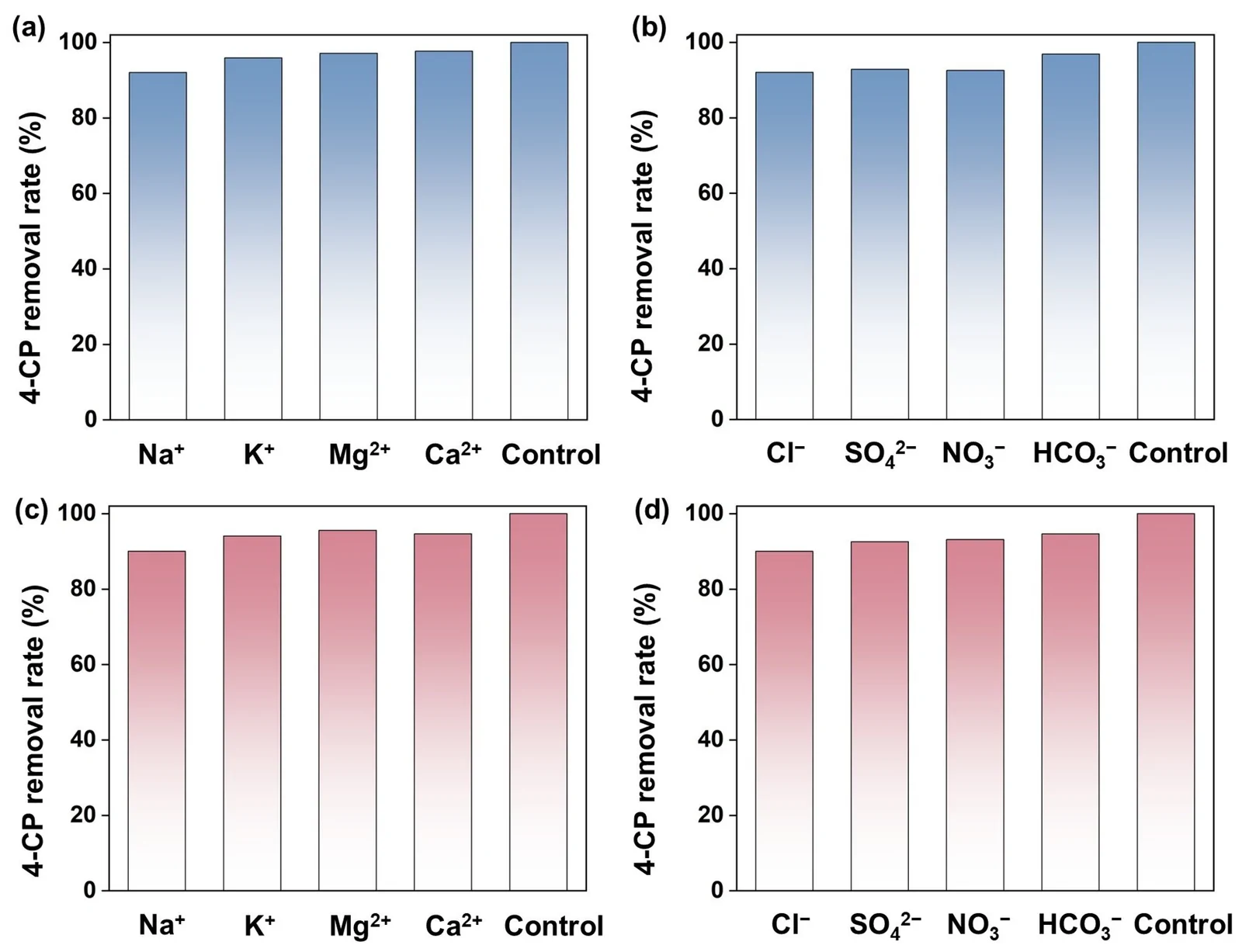
The effects of cations (**a**,**c**) and (**b**,**d**) anions on the degradation of 4-CP in the (**a**,**b**) Co-FeS_2_/PDS and (**c**,**d**) Co-FeS_2_/PMS systems. The concentrations of the co-existing ions were 5 mM.

Inorganic ions present in groundwater may inhibit pollutant degradation via persulfate activation [54]. We further investigated the potential effects of common coexisting ions on the 4-CP degradation efficiency of the Co-FeS_2_/PMS and Co-FeS_2_/PDS systems (Figure 7). The experimental results indicate that common cations (Na^+^, K^+^, Ca^2+^, Mg^2+^) exerted a slight inhibitory effect on the degradation kinetics of 4-CP in both systems; however, the 4-CP removal rate remained over 90% at the end of the 20 min reaction. This inhibitory effect can be attributed to the fact that high concentrations of cations in the aqueous phase can occupy reactive sites on the Co-FeS_2_ surface through surface complexation, creating steric hindrance that impedes effective contact between oxidant molecules and the metal catalytic centers [55]. Moreover, alkaline earth metal ions (especially Ca^2+^ and Mg^2+^) readily undergo hydrolysis or form trace precipitates at local microinterfaces, further masking the electron transfer sites on the material surface [56]. Anions commonly present in groundwater (Cl^−^, SO_4_^2−^, NO_3_^−^, HCO_3_^−^) also exhibited moderate degrees of inhibition on 4-CP degradation. Specifically, the inhibition induced by Cl^−^, NO_3_^−^, and HCO_3_^−^ primarily stems from their ability to compete with the target contaminant, 4-CP, for the •OH or SO_4_^•−^ radicals generated within the systems [57,58]. Although this process is accompanied by the formation of secondary reactive intermediates such as chlorine radicals, dichlorine radical anions, carbonate radicals, and nitrate radicals [57,58], the redox potentials of these secondary radicals are significantly lower than those of the •OH and SO_4_^•−^ radicals. This downgrading of active species manifests macroscopically as a decrease in the 4-CP degradation rate. Regarding the effects of SO_4_^2−^, although its moderate surface pre-accumulation can accelerate the generation of Fe^IV^=O, excessive concentrations of SO_4_^2−^ induce the formation of ion pairs (FeSO_4_), which exhibit poor efficacy in activating persulfates and low iron cycle efficiency, decreasing the homogeneous reactions [54,59]. Additionally, from a thermodynamic perspective, high concentrations of SO_4_^2−^ can lower the reduction potential of SO_4_^•−^ [54,59]. Nevertheless, the final removal rate exceeded 90% in the presence of all the ions, demonstrating the exceptional resilience and environmental adaptability of this catalytic system.

## 4. Conclusions

In this study, Co-FeS_2_ nanomaterials were employed to activate PDS and PMS for the degradation of 4-CP, a model organic pollutant. Although both systems achieved highly efficient degradation of 4-CP, their kinetic patterns were strikingly different. Notably, in contrast to the steady degradation kinetics observed in the Co-FeS_2_/PMS system, the Co-FeS_2_/PDS system exhibited a unique “three-stage” characteristic, wherein the 4-CP degradation rate underwent a significant jump during the 3–5 min phase. This abrupt kinetic transition is attributed to differences in the generation mechanisms of Fe^IV^=O in the two systems. Distinct from the steady generation of Fe^IV^=O in the Co-FeS_2_/PMS system, the Co-FeS_2_/PDS system experienced an initial lag phase followed by a rapid burst of Fe^IV^=O within this specific timeframe. Theoretical calculations further revealed that the underlying mechanism lies in the pre-accumulation of SO_4_^2−^ on the material surface during the initial stage. This pre-accumulation significantly lowered the energy barrier for Fe^IV^=O formation, thereby thermodynamically driving the rapid generation of abundant Fe^IV^=O in the Co-FeS_2_/PDS system after 3 min of reaction. Furthermore, •OH and SO_4_^•−^ also played critical synergistic roles in the degradation processes of both systems; however, •OH concentration is higher than SO_4_^•−^ in the Co-FeS_2_/PDS system, whereas the Co-FeS_2_/PMS system exhibited the reverse trend. Moreover, homogeneous persulfate activation mediated by dissolved Fe(II) contributed to the overall 4-CP degradation, but to different degrees in the two systems. Finally, performance evaluation against diverse background cations and anions verified the robust anti-interference capability of the Co-FeS_2_/persulfates system. These findings offer theoretical support for optimizing persulfate-based groundwater remediation strategies. Future work is warranted to systematically evaluate the system’s performance stability and conduct comprehensive ecotoxicity assessments regarding potential metal leaching (e.g., cobalt) and degradation by-products, thereby further validating the practical applicability and environmental compatibility of the proposed system.

## Data Availability

The original contributions presented in this study are included in the article. Further inquiries can be directed to the corresponding authors.

## Supplementary Materials

The following supporting information can be downloaded at: https://www.mdpi.com/article/10.3390/toxics14080663/s1, Figure S1: (a) TEM and (b) HRTEM images of Co-FeS_2_.; Figure S2: XRD pattern of Co-FeS_2_; Figure S3. Degradation curves (a) and kinetic fitting curves (b) of benzoic acid (BA) and p-nitrobenzoic acid (p-NBA) in the Co-FeS_2_/PDS system; Figure S4: Degradation curves (a) and kinetic fitting curves (b) of benzoic acid (BA) and p-nitrobenzoic acid (p-NBA) in the Co-FeS_2_/PMS system; Table S1: Conditions for liquid chromatography analysis; Table S2: XPS fitting parameters of Fe 2p of Co-FeS_2_ after the 20 min reaction; Table S3: XPS fitting parameters of Co 2p of Co-FeS_2_ after the 20 min reaction; Table S4: XPS fitting parameters of S 2p of Co-FeS_2_ after the 20 min reaction.

## References

[1] Zhao B., Huang F.Y., Zhang C., Huang G.X., Xue Q., Liu F. (2020). Pollution characteristics of aromatic hydrocarbons in the groundwater of China. J. Contam. Hydrol..

[2] Li Y., Huo Z., Ying Y., Duan L., Jiang C., Chen W. (2024). Effects of transient flow conditions on colloid-facilitated release of decabromodiphenyl ether: Implications for contaminant mobility at e-waste recycling sites. Eco-Environ. Health.

[3] Wang Y., Li G., Li X., Yang Y., Ding K., Xing S., Zhang Y., Zhang L. (2024). Human Health Risk Assessment of Chlorinated Hydrocarbons in Groundwater Based on Multi-Pathway Analysis. Toxics.

[4] Xu J., Li H., Lowry G.V. (2021). Sulfidized Nanoscale Zero-Valent Iron: Tuning the Properties of This Complex Material for Efficient Groundwater Remediation. Acc. Mater. Res..

[5] Liang Z.S., Jiang C.J., Li Y.Y., Liu Y.Q., Yu J.G., Zhang T., Alvarez P.J.J., Chen W. (2024). Single-Atom Iron Can Steer Atomic Hydrogen toward Selective Reductive Dechlorination: Implications for Remediation of Chlorinated Solvents-Impacted Groundwater. Environ. Sci. Technol..

[6] Liu L., Meng Y., Zhang Q., Sun Y., Yu M., Zheng M. (2025). Silicified microscale zero-valent iron for in situ remediation of chloramphenicol in groundwater: Reactivity coupling with mobility. Chem. Eng. J..

[7] Yu M.H., Yang J.Y., Chen J.S., Luo H.Y., Ma H., Pu S.Y. (2025). Enhancement of 2,4,6-trichlorophenol degradation in groundwater via natural pyrite-activated percarbonate: Mechanistic insights and efficiency evaluation. J. Environ. Chem. Eng..

[8] Duan Y., Liu J., Liu Q., Li T., Zhao H., Shen Z. (2026). Remediation of characteristic contaminants in groundwater of chemical industrial by the activation of PMS: Recent developments and challenges-a mini-review. Chin. Chem. Lett..

[9] Zhang C., Xin S., Zhao W., Hong Z., Qin C. (2025). A novel technology based on aeration-coupled molecular oxygen activation for in-situ sustainable groundwater remediation: Mechanisms and feasibility assessment. Water Res..

[10] Jin X., Wu D., Ling J., Wang C., Liu C., Gu C. (2019). Hydrolysis of Chloramphenicol Catalyzed by Clay Minerals under Nonaqueous Conditions. Environ. Sci. Technol..

[11] Li T., Zhong W., Jing C., Li X., Zhang T., Jiang C., Chen W. (2020). Enhanced Hydrolysis of p-Nitrophenyl Phosphate by Iron (Hydr)oxide Nanoparticles: Roles of Exposed Facets. Environ. Sci. Technol..

[12] Jin X., Wu D.D., Liu C., Huang S.H., Zhou Z.Y., Wu H., Chen X.R., Huang M.Y., Zhou S.D., Gu C. (2022). Facet effect of hematite on the hydrolysis of phthalate esters under ambient humidity conditions. Nat. Commun..

[13] Chen X., Zhang S., Yi L., Liu Z., Ye X., Yu B., Shi S., Lu X. (2022). Evaluation of Biodegradation of BTEX in the Subsurface of a Petrochemical Site near the Yangtze River, China. Int. J. Environ. Res. Public Health.

[14] Pilloni G., Wilson J., Rosolina S., Oyston B., Taggart D., Key T.A. (2025). Field-Scale qPCR Data to Estimate Rate Constants for Toluene Biodegradation in Groundwater. Groundw. Monit. Rem..

[15] Zhang T., Zheng W., Zhang N., Wei T.H., Liu Y.Y. (2026). Efficient in situ chemical oxidation of petroleum hydrocarbons in soil by foaming percarbonate. Chem. Eng. J..

[16] Yang X.Y., Cai J.S., Wang X.N., Li Y.F., Wu Z.X., Wu W.D., Chen X.D., Sun J.Y., Sun S.P., Wang Z.H. (2020). A Bimetallic Fe-Mn Oxide-Activated Oxone for In Situ Chemical Oxidation (ISCO) of Trichloroethylene in Groundwater: Efficiency, Sustained Activity, and Mechanism Investigation. Environ. Sci. Technol..

[17] He F.R., Xu L.R., Wang H.Y., Jiang C.J. (2024). Recent Progress in Molecular Oxygen Activation by Iron-Based Materials: Prospects for Nano-Enabled In Situ Remediation of Organic-Contaminated Sites. Toxics.

[18] Li Y.T., Sui Q., Li X., Wang Y.Q., Liu X.Y., Liu H., Du W.Y. (2024). Enhanced in-situ zero-valent iron activated persulfate oxidation with electrokinetics for the remediation of petroleum hydrocarbon contaminated soil. J. Environ. Chem. Eng..

[19] Chen W., Cui P., Zhou L., Zheng H., Zhao X.D., Liu G.S., Zhang J.T., Zhong H. (2025). In-situ activation of persulfate by emplaced magnetite nanoparticles for degradation of 1,2-dichloroethane in porous media. Water Res..

[20] Wei W., Cao W., Qu R., Wang Z. (2026). Metal-Modified Biochar Activates Persulfate for the Removal of Phenolic Pollutants from Water: Mechanism Prediction and Non-Radical Targeted Regulation. Toxics.

[21] Liang C., Liu X.F., Ling C.C., Guo F.R., Li M.Q., Zhang X., She Y.Y., Sun H.W., Ai Z.H., Zhang L.Z. (2024). Proton-coupled electron transfer activation of peroxydisulfate with phosphorylated zero-valent iron. Appl. Catal. B-Environ..

[22] Jiang X.H., Zhou B.H., Yang W.J., Chen J.Y., Miao C., Guo Z.Y., Li H., Hou Y., Xu X.H., Zhu L.Z. (2024). Precise coordination of high-loading Fe single atoms with sulfur boosts selective generation of nonradicals. Proc. Natl. Acad. Sci. USA.

[23] Wang F., Zhou Y., Weng X., Wang L., Gao S., Zhao Y., Zhan W., Guo Y., Dai Q. (2025). Oxygen Vacancies Engineering of Co_3_O_4_ to Modulate the Adjacent Environment: Boosted Singlet Oxygen Generation in Peroxymonosulfate-Mediated 2-Chlorophenol Degradation. Environ. Sci. Technol..

[24] Tu S.C., Chen T., Zhang J.H., Huang H.W., Yan B. (2025). Heterogeneous AOP degradation of mineral flotation reagents (MFRs): A critical review of charge migration, interfacial reaction and transformation path. Chem. Eng. J..

[25] Long X.H., Li D.Z., Huang R.F., Huang S.Y., Zhang S.Y. (2024). Insights of Electrocatalytic Oxidation Mechanisms Utilizing Different Oxidants for Degradation of Organic Pollutants: Option for Sustainability. ACS Sustain. Chem. Eng..

[26] Song H.R., Yan L.X., Wang Y.W., Jiang J., Ma J., Li C.P., Wang G., Gu J., Liu P. (2020). Electrochemically activated PMS and PDS: Radical oxidation versus nonradical oxidation. Chem. Eng. J..

[27] Shang Y.A., Kan Y.J., Xu X. (2023). Stability and regeneration of metal catalytic sites with different sizes in Fenton-like system. Chin. Chem. Lett..

[28] Li Y.T., Zhao B.X., Ying G.G., Wu D.L., Shih K.M., Feng Y. (2025). Activation of peroxymonosulfate by atomically Dispersed FeN6 Sites: Formation of singlet oxygen and mechanisms. Sep. Purif. Technol..

[29] Wu M., Sun W., Meng X., Kang J., Yang Y. (2023). Peroxymonosulfate Activation by Iron Sulfide Minerals for Degradation of Various Thiol Collectors in Mineral Processing Wastewater: Performance, Mechanism, and Structure–Activity Relationship. ACS ES&T Water.

[30] Su L., Li Y.F., Wang Z.K., Lou Y.Y., Zheng Q.Z., Wu Z.X., Sun S.P. (2023). Nonradical activation of Oxone over Fe-doped nitrogen carbon (Fe@NC) armor catalysts for efficient degradation of anthropogenic phenolics (p-nitrophenol, 4-chlorophenol) in groundwater matrices. Chem. Eng. J..

[31] Ji Y.F., Wang L., Jiang M.D., Lu J.H., Ferronato C., Chovelon J.M. (2017). The role of nitrite in sulfate radical-based degradation of phenolic compounds: An unexpected nitration process relevant to groundwater remediation by in-situ chemical oxidation (ISCO). Water Res..

[32] Liang Y., Tan X., Tu Z., Mo S., He C., Liang X., Dang Z. (2026). Cobalt doping enhances the catalytic activity of pyrite: Efficient degradation of sulfamethazine via synergistic oxidative pathways. Chem. Eng. J..

[33] He F.R., Shen M.M., Liang Z.S., Low J., Zhang T., Chen W., Jiang C.J. (2025). Cobalt doping of FeS_2_ simultaneously promotes radical and nonradical pathways of O_2_ activation by creating dual active sites. Environ. Sci.-Nano.

[34] Gu Q., Gao X., Liang B., Liu X., Wang J., Guo T., Zhu W., Luo Y. (2025). Overlooked role of transition metal impurities (cobalt and nickel) substitution in tuning pyrite to activate peroxymonosulfate for degradation of emerging pollutants. Sep. Purif. Technol..

[35] Zhang K., Park M., Zhou L.M., Lee G.H., Shin J., Hu Z., Chou S.L., Chen J., Kang Y.M. (2016). Cobalt-Doped FeS_2_ Nanospheres with Complete Solid Solubility as a High-Performance Anode Material for Sodium-Ion Batteries. Angew. Chem. Int. Ed..

[36] Gong W.W., He D.D., Wang X., Yan Y.T., Dionysiou D.D., Blaney L., Peng G.L. (2024). The role of Fe(IV) in the zero-valent iron biochar activated persulfate system for treatment of contaminants of emerging concern. Chem. Eng. J..

[37] Hong W.J., Zou J.M., Zhao M.Z., Yan S.W., Song W.H. (2024). Development of a Five-Chemical-Probe Method to Determine Multiple Radicals Simultaneously in Hydroxyl and Sulfate Radical-Mediated Advanced Oxidation Processes. Environ. Sci. Technol..

[38] Deniere E., Alagappan R.P., Van Langenhove H., Van Hulle S., Demeestere K. (2022). The ozone-activated peroxymonosulfate process (O_3_/PMS) for removal of trace organic contaminants in natural and wastewater: Effect of the (in)organic matrix composition. Chem. Eng. J..

[39] Lee D., Lee J., Yu G., Kim K., Kim J., Mok D.H., Jang A., Jung M., Ahn H., Back S. (2025). Enhanced Fe^IV^=O Generation via Peroxymonosulfate Activation by an Edge-Site Engineered Single-Atom Iron Catalyst. Small.

[40] Zou Y.X., Fu S.H., Xu Z.Y., Zhou X.C., Li J., Zhang Y.Y., Lu Y., Li S.Y., Zhang T.T. (2025). Unveiling the long-range interaction of sulfur in the second shell of Fe-N4 single-atom sites for highly selective generation of high-valent iron-oxo species in peroxymonosulfate activation. Chem. Eng. J..

[41] Jiang L.B., Hu Z.X., Xie S.R., Shangguan Z.C., Lu Q.M., Chen H.Y., Guo J.Y., Li J.Y., Zhao W., Wang H. (2026). Axial oxygen-coordinated asymmetric structure Fe-N_4_O_1_ enhanced non-radical pathway for ultrafast degradation. Appl. Catal. B-Environ..

[42] Wang X., Gong W., Zhu J., Peng G. (2024). New insights into the quantiffcation of Fe(IV) using methyl phenyl sulfoxide (PMSO) as probe in the iron-based heterogeneous catalyst activated persulfate process. Environ. Pollut..

[43] Lei Y., Yu Y.F., Lei X., Liang X., Cheng S.S., Ouyang G.F., Yang X. (2023). Assessing the Use of Probes and Quenchers for Understanding the Reactive Species in Advanced Oxidation Processes. Environ. Sci. Technol..

[44] Zong Y., Shao Y.F., Zeng Y.Q., Shao B.B., Xu L.Q., Zhao Z.Y., Liu W., Wu D.L. (2021). Enhanced Oxidation of Organic Contaminants by Iron(II)-Activated Periodate: The Significance of High-Valent Iron-Oxo Species. Environ. Sci. Technol..

[45] Wang Z., Qiu W., Pang S.Y., Gao Y., Zhou Y., Cao Y., Jiang J. (2020). Relative contribution of ferryl ion species (Fe(IV)) and sulfate radical formed in nanoscale zero valent iron activated peroxydisulfate and peroxymonosulfate processes. Water Res..

[46] Li T.Z., Wang X.Y., Fan Z.H., Wang X.N., Wu Z.H., Akram M., Wang L., Liu J., Pan J.W., Gao B.Y. (2024). Unique role of doped Mo into Fe-based catalyst to intensify peroxydisulfate activation for micropollutants degradation: Promote the conversion of SO_4_^·-^ to Fe^IV^=O. Chem. Eng. J..

[47] Gao Y., Champagne P., Blair D., He O., Song T. (2020). Activated persulfate by iron-based materials used for refractory organics degradation: A review. Water Sci. Technol..

[48] Bonnissel-Gissinger P., Alnot M., Ehrhardt J.-J., Behra P. (1998). Surface Oxidation of Pyrite as a Function of pH. Environ. Sci. Technol..

[49] Pignatello J.J., Oliveros E., MacKay A. (2007). Advanced oxidation processes for organic contaminant destruction based on the Fenton reaction and related chemistry. Crit. Rev. Environ. Sci. Technol..

[50] Stumm W., Morgan J.J. (1996). Aquatic Chemistry: Chemical Equilibria and Rates in Natural Waters.

[51] Li Q., Jiao Q.Z., Yan Y., Li H.J., Zhou W., Gu T.T., Shen X.R., Lu C.X., Zhao Y., Zhang Y.Y. (2022). Optimized Co-S bonds energy and confinement effect of hollow MXene@CoS2/NC for enhanced sodium storage kinetics and stability. Chem. Eng. J..

[52] Nesbitt H.W., Muir I.J. (1994). X-ray photoelectron spectroscopic study of a pristine pyrite surface reacted with water vapour and air. Geochim. Cosmochim. Acta.

[53] Fan J., Gu L., Wu D., Liu Z. (2018). Mackinawite (FeS) activation of persulfate for the degradation of p-chloroaniline: Surface reaction mechanism and sulfur-mediated cycling of iron species. Chem. Eng. J..

[54] Luo Y., Zhou Y., Xu T., Su R., Ma X., Yan W. (2026). Flower-like CoFe-LDH Activated Peroxymonosulfate for Tetracycline Degradation: Efficiency and Mechanism. Toxics.

[55] Dzombak D.A., Morel F.M.M. (1990). Surface Complexation Modeling: Hydrous Ferric Oxide.

[56] Wang J.H., Kong F.Y., Liu B.F., Zhuo S.N., Ren N.Q., Ren H.Y. (2024). Enhanced photocatalytic degradation of diclofenac by UiO-66/MgAl-LDH: Excellent performances and mechanisms. Environ. Sci.-Nano.

[57] Wacławek S., Lutze H.V., Grübel K., Padil V.V.T., Černík M., Dionysiou D.D. (2017). Chemistry of persulfates in water and wastewater treatment: A review. Chem. Eng. J..

[58] Cao J., Lai L., Lai B., Yao G., Chen X., Song L. (2019). Degradation of tetracycline by peroxymonosulfate activated with zero-valent iron: Performance, intermediates, toxicity and mechanism. Chem. Eng. J..

[59] Tang C., Long Z., Wang Y., Ma D., Zhu X. (2022). Sulfate Decelerated Ferrous Ion-Activated Persulfate Oxidation of Azo Dye Reactive Brilliant Red: Influence Factors, Mechanisms, and Control Methods. Catalysts.

