# Supplementary material for: Persulfate Activation by Cobalt-Doped Pyrite Nanoparticles for Oxidative Removal of 4-Chlorophenol
Source: Toxics. 2026 Jul 27;14(8):663. doi: 10.3390/toxics14080663 (PMC13517888; doi:10.3390/toxics14080663)
Supplement: Supplementary file 1 [file toxics-14-00663-s001.zip › toxics-4408280-supplementary.pdf]

Supplementary Material

Persulfate Activation by Cobalt-Doped Pyrite Nanoparticles for Oxidative Removal of 4-Chlorophenol

Mengyang Ni [1], Fangru He [2,*], Chuanjia Jiang [2,*], and Hongyang Wang [1]

[1] State Key Laboratory of Environmental Criteria and Risk Assessment, Chinese Research Academy of Environmental Sciences, Beijing 100012, China

[2] College of Environmental Science and Engineering, Nankai University, Tianjin 300350, China

* Corresponding authors: Fangru He (1120220318@main.nankai.edu.cn); Chuanjia Jiang (jiangcj@nankai.edu.cn)

Number of pages: 15

Number of figures: 4

Number of tables: 4

Chemicals

Ferrous sulfate heptahydrate ($FeSO_4 \cdot 7H_2O$, 99.99%), cobaltous sulfate heptahydrate ($CoSO_4 \cdot 7H_2O$, 99.99%), urea (99.5%), *N,N*-dimethylformamide (99.5%), ethylene glycol (99.0%), 4-chlorophenol (4-CP, 99.5%), peroxymonosulfate (PMS, 47%), peroxydisulfate (PDS, 98%), methyl phenyl sulfoxide (PMSO, 99.0%), methyl phenyl sulfone ($PMSO_2$, 99.0%), benzoic acid (BA, 99.5%)), *p*-nitrobenzoic acid (*p*-NBA, 99.5%), sodium thiosulfate (94%), methanol (MeOH, 99.9%), and tert-butyl alcohol (TBA, 99.8%) were acquired from Aladdin Corporation (Shanghai, China). Sublimed sulfur (S, 99.99%) was purchased from Alfa Aesar Corporation (Shanghai, China). Deionized water (18.2 MΩ·cm) from a Millipore water purification system was used throughout the experiments. All chemicals and reagents were used without further purification.

Preparation and Characterization of Materials

Cobalt-doped FeS_2 (Co-FeS_2) was synthesized via a solvothermal method [1,2]. Briefly, 1.9 mmol of $FeSO_4 \cdot 7H_2O$, 0.1 mmol of $CoSO_4 \cdot 7H_2O$, and 10 mmol of urea were initially dissolved in a mixed solvent comprising 30 mL of *N,N*-dimethylformamide and 40 mL of ethylene glycol. Subsequently, 25 mmol of sublimed sulfur was added to the solution. After stirring for 1 h, the suspension was transferred to a 100 mL Teflon-lined stainless-steel autoclave, which was then tightly sealed and maintained at 180 °C for 12 h. The resulting product was centrifuged and thoroughly washed with deionized water and absolute ethanol. The black solid was then dried for 12 h in a freeze dryer. The materials were characterized by X-ray diffraction (XRD, Ultima IV, Rigaku, Japan) and transmission electron microscopy (TEM, JEOL JEM-2800, Japan).

Persulfate Activation and 4-CP Degradation Experiment

Typically, 2 mL of a 0.1 mM 4-CP solution and 7.9 mL of H_2O were added to a 25 mL

conical flask. After adjusting the pH to approximately 5.8 using 1 M NaOH or 1 M H_2SO_4, 100 μL of borate buffer was added, and the pH was adjusted to 7.2. Then, a certain amount (2 or 4 mg) of Co-FeS_2 was added, and the suspension was designated as Suspension A. Meanwhile, 2 mL of PMS or PDS solution and 8 mL of water were added to a 25 mL beaker, and the pH was adjusted to approximately 5.8. Instead of using identical doses, 10 mM PDS and 1 mM PMS were selected to deliver an equivalent overall degradation of 4-CP within a similar time frame. Then, 100 μL of borate buffer was added to adjust the pH to 7.2, and this solution was designated as Solution B. Next, Solution B was quickly poured into Suspension A, initiating the reaction. At the scheduled sampling time, 0.2 mL of the reaction mixture was added to an equal volume of sodium thiosulfate solution. The quenched reaction mixture was filtered through a 0.22 μm disposable syringe filter into a 2 mL liquid-phase injection vial for determination of the 4-CP concentration. The entire degradation experiment was conducted on a constant-temperature (25 °C) shaking incubator at 180 rpm. Three parallel experiments were conducted for each group.

The procedure for the quenching experiment was identical to that of the degradation experiment, except that the inhibitors, MeOH and TBA [3], were added to the conical flask.

For the homogeneous persulfate activation experiments mediated by dissolved iron ions, $FeSO_4 \cdot 7H_2O$ was used as the iron source instead of Co-FeS_2. Its concentration was set to match the total dissolved iron concentration in each system at 20 min. To ensure that the iron ions remained in a dissolved state, the reaction pH was set to 3, and the remaining steps were identical to those described above.

The experiments on the effects of co-existing inorganic ions were performed using procedures that were essentially the same as those described for the degradation experiment. The only difference was that a solution of the inorganic ion was added to the conical flask before the

reaction was initiated. The concentrations of co-existing ions were all set to 5 mM.

The concentration of 4-CP was determined using high-performance liquid chromatography (HPLC, Agilent 1260, USA). The test conditions were as follows: Agilent Eclipse Plus-C18 column (5 μm, 150 × 4.6 mm I.D.), flow rate of 1.0 mL/min, column temperature maintained at 30 °C, injection volume of 100 μL, mobile phase consisting of acetonitrile and 0.1% formic acid in a 70:30 volume ratio, UV detector with a detection wavelength of 280 nm.

Detection and Quantification of Reactive Species

The steady-state concentrations of •OH and $SO_4^{•-}$ were calculated using competitive kinetic experiments. BA and *p*-NBA were used as probes. The calculation of the steady-state concentrations was performed according to Equations S1 to S4:

Rate laws of BA and *p*-NBA degradation by •OH and $SO_4^{•-}$:

$$-\frac{d[\text{BA}]}{\text{d}t} = k_{\text{BA},\bullet\text{OH}}[\bullet\ \text{OH}][\text{BA}] + k_{\text{BA},\text{SO}_4^{\bullet-}}[\text{SO}_4^{\bullet-}]\ [\text{BA}] \quad \text{(S1)}$$

$$-\frac{d[p\text{-NBA}]}{\text{d}t} = k_{p\text{-NBA},\bullet\text{OH}}[\bullet\ \text{OH}][p\text{-NBA}] + k_{p\text{-NBA},\text{SO}_4^{\bullet-}}[\text{SO}_4^{\bullet-}]\ [p\text{-NBA}] \quad \text{(S2)}$$

Integral equations:

$$-\ln\frac{[\text{BA}]}{[\text{BA}]_0} = (k_{\text{BA},\bullet\text{OH}}[\bullet\ \text{OH}]_{\text{ss}} + k_{\text{BA},\text{SO}_4^{\bullet-}}[\text{SO}_4^{\bullet-}]_{\text{ss}})t = k_{\text{obs,BA}}t \quad \text{(S3)}$$

$$-\ln\frac{[p\text{-NBA}]}{[p\text{-NBA}]_0} = (k_{p\text{-NBA},\bullet\text{OH}}[\bullet\ \text{OH}]_{\text{ss}} + k_{p\text{-NBA},\text{SO}_4^{\bullet-}}[\text{SO}_4^{\bullet-}]_{\text{ss}})t = k_{\text{obs},p\text{-NBA}}t \quad \text{(S4)}$$

The apparent first-order reaction rate constants (k_{obs}, BA and $k_{obs,p\text{-NBA}}$) can be obtained by fitting the curves of –ln[BA]/$[BA]_0$ and –ln[*p*-NBA]/$[p\text{-NBA}]_0$, respectively, as a function of time (*t*). The rate constants for the reactions of BA with •OH and $SO_4^{•-}$ are 5.8×10^9 and 1.2×10^9 M^{-1} s^{-1}, respectively [3]. The rate constants for the reactions of *p*-NBA with •OH and $SO_4^{•-}$ are 2.6×10^9 and 1×10^6 M^{-1} s^{-1}, respectively [4]. The concentrations of BA and *p*-NBA were determined using HPLC, and the test conditions are listed in Table S1.

PMSO was used as a probe for determination of $Fe^{IV}=O$ generation, and the formation of $Fe^{IV}=O$ was evaluated by calculating the efficiency of PMSO conversion to $PMSO_2$. In the typical experiment, 5 mL of a 0.1 mM probe solution and 4.9 mL of H_2O were added to a 25 mL conical flask. After adjusting the pH to approximately 5.8 using 1 M NaOH or 1 M H_2SO_4, 100 μL of borate buffer was added, and the pH was further adjusted to 7.2. Then, the Co-FeS_2 was added, and this suspension was designated as Suspension C. Concurrently, 2 mL of the oxidant solution and 8 mL of water were mixed in a 25 mL beaker, and the pH was adjusted to 7.2 using the same acid/base and borate buffer approach; this was designated as Solution D. The reaction was initiated by rapidly adding Solution D into Suspension C. At predetermined intervals, 0.2 mL of the reaction mixture was added to an equal volume of sodium thiosulfate solution. The quenched reaction mixture was filtered through a 0.22 μm disposable syringe filter into a 2 mL liquid-phase injection vial for the determination of the probe or its product concentration by HPLC, with the test conditions for the HPLC analysis listed in Table S1.

Determination of Fe(II) and Total Fe Concentrations in Solution

The Fe(II) concentration in the solution was determined using the 1,10-phenanthroline colorimetric method. Total iron content was determined by reducing Fe(III) to Fe(II) using hydroxylamine hydrochloride. The specific experimental procedure is as follows: At a predetermined time point, 0.3 mL of the reaction mixture was added to 0.3 mL of sodium thiosulfate solution to stop the reaction. After filtration through a 0.22 μm filter membrane, 0.5 mL of the sample solution was transferred to a 5 mL centrifuge tube, and 1 mL of acetic acid-sodium acetate buffer solution and 2 mL of 1,10-phenanthroline color reagent were added sequentially. For the determination of total iron, 1 mL of hydroxylamine hydrochloride was added, and the solution was mixed well and allowed to stand for 10 min for color development.

Subsequently, the absorbance at a wavelength of 510 nm was measured using a UV–visible spectrophotometer (Agilent Cary 100 G9821A, USA).

Density Functional Theory (DFT) Calculation

This study employs first-principles periodic DFT calculations, which were performed using the Vienna ab initio Simulation Program (VASP). All calculations were performed using the Projector-Augmented Wave (PAW) method, with the plane-wave cutoff set at 450 eV. Partial occupation of the Kohn–Sham orbitals was allowed via the Gaussian broadening method (with a broadening width of 0.05 eV). Electronic self-consistency iterations were considered converged when the energy change was less than 10^{-5} eV. The convergence criterion for geometric optimization was set to an interaction force change of less than 0.03 eV/Å. The Brillouin zone was sampled using a 2 × 2 × 1 Monkhorst-Pack-type K-point grid. The (200) crystal plane was selected as the model surface because XRD results indicated that the (200) crystal plane was the predominant exposed facet for Co-FeS_2 [2]. Two models were constructed for comparison: one pristine surface model, and the other with a pre-adsorbed SO_4^{2-}, designed to elucidate the influence of sulfate adsorption on PDS activation.

The Gibbs free energy change (ΔG) was calculated using Equation S5:

$$\Delta G = \Delta E + \Delta E_{ZPE} - T\Delta S \qquad \text{(S5)}$$

where ΔE, ΔE_{ZPE}, and $T\Delta S$ represent the ground-state energy, the zero-point energy difference, and the entropy contribution, respectively, with the temperature T set to 300 K.

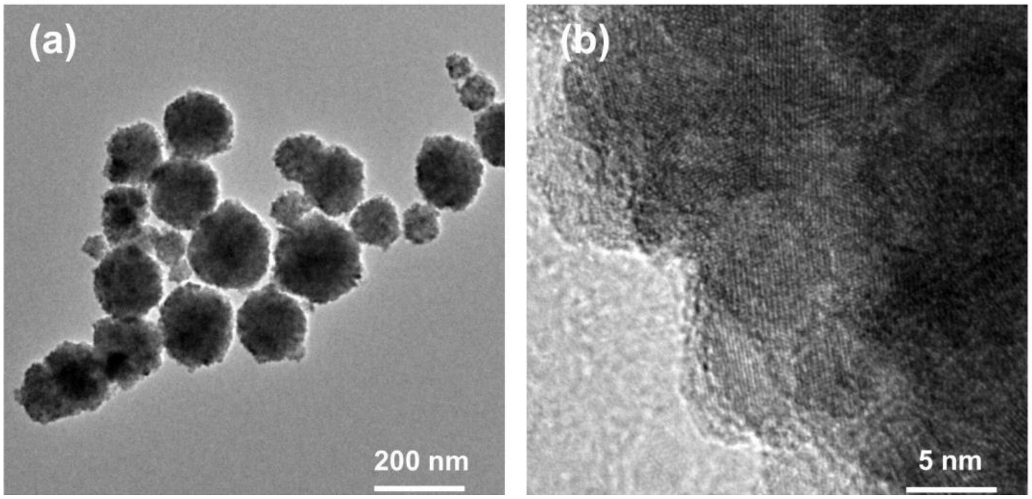

Figure S1. (a) TEM and (b) HRTEM images of Co-FeS_2.

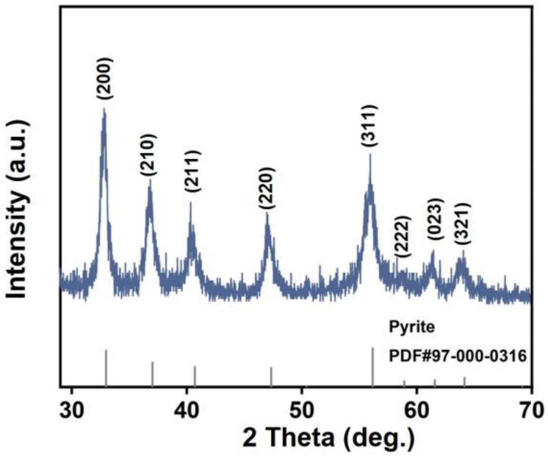

Figure S2. XRD pattern of Co-FeS_2.

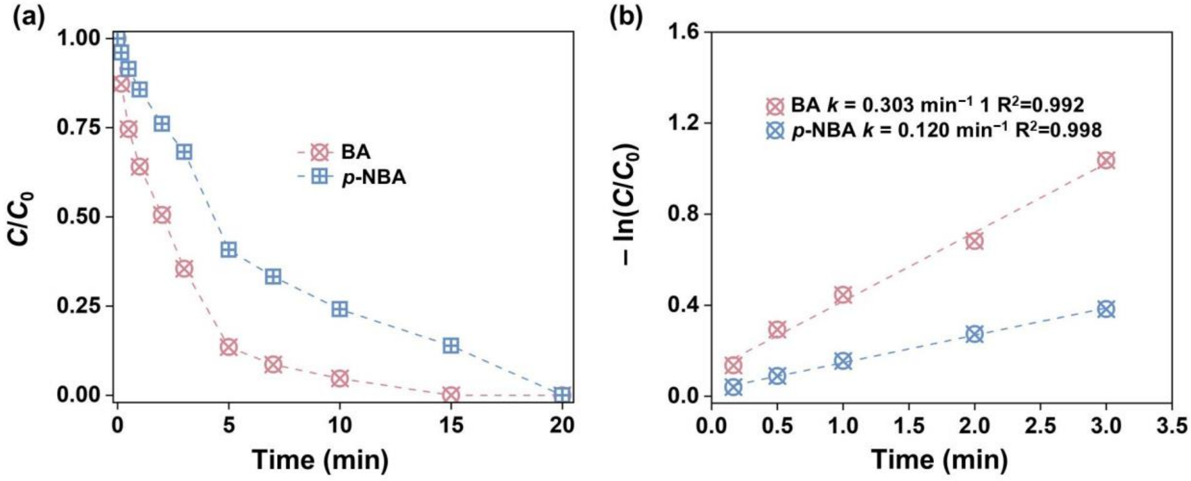

Figure S3. Degradation curves (a) and kinetic fitting curves (b) of benzoic acid (BA) and *p*-nitrobenzoic acid (*p*-NBA) in the Co-FeS_2/PDS system.

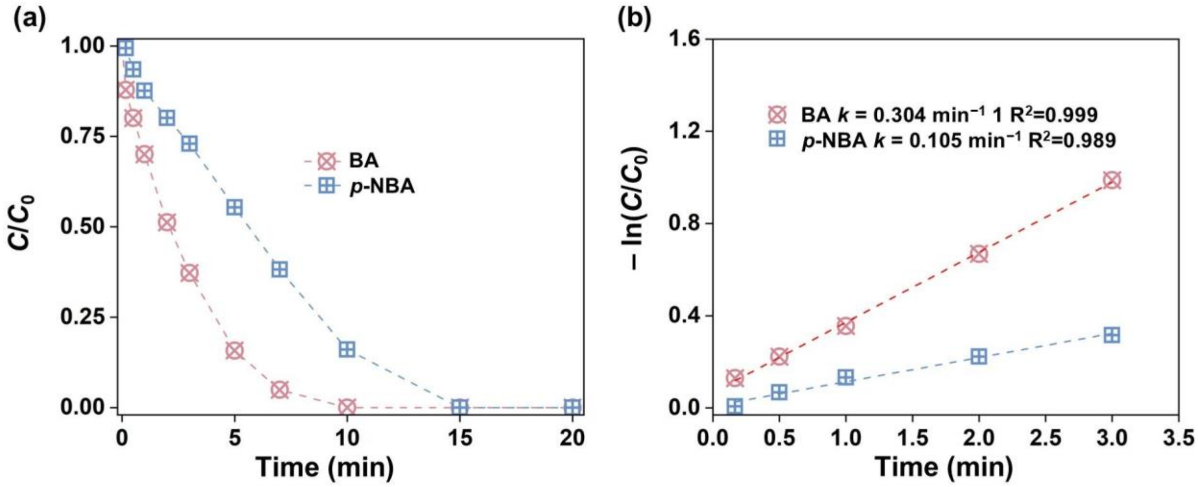

Figure S4. Degradation curves (a) and kinetic fitting curves (b) of benzoic acid (BA) and p-nitrobenzoic acid (*p*-NBA) in the Co-FeS_2/PMS system.

Table S1. Conditions for Liquid Chromatography Analysis.

Probe/ product	Flow rate (mL/min)	Detector wavelength (nm)	Mobile phase
BA	1	280	Methanol:H_2O = 85:15（v/v）
p-NBA	1	255	Acetonitrile:0.1%Formic acid = 65:35（v/v）
PMSO	1	217	Acetonitrile:H_2O = 30:70（v/v）
$PMSO_2$	1	217	Acetonitrile:H_2O = 30:70（v/v）

Table S2. XPS fitting parameters of Fe 2p of Co-FeS_2 after the 20 min reaction.

Sample	Orbitals	Bind energy (eV)	FWHM (eV)	Area (CPS eV^{-1})	Assignment	Atomic (%)
Co-FeS_2/PDS	Fe $2p_{3/2}$	706.8	1.7	39505	Fe(II)	68.7
	Fe $2p_{1/2}$	719.6	2.2	19752		
	Fe $2p_{3/2}$	709.6	3.5	19983	Fe(III)	31.3
	Fe $2p_{1/2}$	722.7	3.5	6994		
Co-FeS_2/PMS	Fe $2p_{3/2}$	707.2	1.7	36159	Fe(II)	68.2
	Fe $2p_{1/2}$	720.0	2.2	18079		
	Fe $2p_{3/2}$	709.9	3.3	18769	Fe(III)	31.8
	Fe $2p_{1/2}$	723.3	3.2	6569		

Notes: FWHM, full width at half maximum; CPS, counts per second.

Table S3. XPS fitting parameters of Co 2p of Co-FeS_2 after the 20 min reaction.

Sample	Orbitals	Bind energy (eV)	FWHM (eV)	Area (CPS eV^{-1})	Assignment	Atomic (%)
Co-FeS_2/PDS	Co $2p_{3/2}$	778.7	2.1	10020	Co(III)	34.2
	Co $2p_{1/2}$	793.7	2.1	4629		
	Co $2p_{3/2}$	782.1	5.7	21647	Co(II)	65.8
	Co $2p_{1/2}$	797.1	5.7	6494		
Co-FeS_2/PMS	Co $2p_{3/2}$	779.1	2.3	8457	Co(III)	28.5
	Co $2p_{1/2}$	794.0	2.3	3797		
	Co $2p_{3/2}$	783.0	5.6	23676	Co(II)	71.5
	Co $2p_{1/2}$	797.4	5.6	7102		

Notes: FWHM, full width at half maximum; CPS, counts per second.

Table S4. XPS fitting parameters of S 2p of Co-FeS_2 after the 20 min reaction.

Sample	Bind energy (eV)	FWHM (eV)	Area (CPS eV^{-1})	Assignment	Atomic (%)
Co-FeS_2/PDS	162	2.5	37658	S(–II)	55.8
	163	2.0	18829	S_n(–II)	36.0
	165	2.0	3669		
	166	2.0	1834		
	168	2.0	3679	S(IV)	8.2
	169	2.0	1839		
Co-FeS_2/PMS	162	2.0	36386	S(–II)	55.6
	163	2.0	18193	S_n(–II)	35.2
	165	2.0	3220		
	166	2.0	1609		
	168	2.0	4031	S(IV)	9.2
	169	2.0	2016		

Notes: FWHM, full width at half maximum; CPS, counts per second.

References

1. Zhang, K.; Park, M.; Zhou, L.M.; Lee, G.H.; Shin, J.; Hu, Z.; Chou, S.L.; Chen, J.; Kang, Y.M. Cobalt-Doped FeS_2 Nanospheres with Complete Solid Solubility as a High-Performance Anode Material for Sodium-Ion Batteries. Angew. Chem., Int. Ed. 2016, 55, 12822–12826.

2. He, F.R.; Shen, M.M.; Liang, Z.S.; Low, J.; Zhang, T.; Chen, W.; Jiang, C.J. Cobalt doping of FeS_2 simultaneously promotes radical and nonradical pathways of O_2 activation by creating dual active sites. Environ. Sci. Nano 2025, 12, 5407–5418.

3. Lei, Y.; Yu, Y.F.; Lei, X.; Liang, X.; Cheng, S.S.; Ouyang, G.F.; Yang, X. Assessing the Use of Probes and Quenchers for Understanding the Reactive Species in Advanced Oxidation Processes. Environ. Sci. Technol. 2023, 57, 5433–5444.

4. Deniere, E.; Alagappan, R.P.; Van Langenhove, H.; Van Hulle, S.; Demeestere, K. The ozone-activated peroxymonosulfate process (O_3/PMS) for removal of trace organic contaminants in natural and wastewater: effect of the (in)organic matrix composition. Chem. Eng. J. 2022, 430, 133000.
